# Assessing the impact of AI-enabled BIM digital twins on construction risks: a Bayesian Network of Jordanian expert beliefs

**DOI:** 10.3389/frai.2026.1846388

**Published:** 2026-08-25

**Authors:** Faten Albtoush, Ja’far A. Aldiabat Al-Btoosh, Taiseer Mustafa Rawashdeh, Mohammed A. K. A. Al-Btoush

**Affiliations:** 1 Faculty of Engineering, Civil Department, Jadara University, Irbid, Jordan; 2 Faculty of Engineering Technology, Zarqa University, Zarqa, Jordan; 3 Faculty of Engineering, Isra University, Amman, Jordan

**Keywords:** artificial intelligence, Bayesian Networks, BIM, construction project risk, decision support, digital twin, expert elicitation, hierarchical Bayesian

## Abstract

Building projects in Jordan routinely experience substantial schedule delays and cost overruns, yet robust empirical evidence on the impacts of Artificial Intelligence (AI) and Building Information Modelling (BIM)-based Digital Twins remains scarce. This study develops a belief-based, probabilistic causal model of how AI-enabled BIM Digital Twin technologies may affect time–cost risks in Jordanian reinforced-concrete building projects. Using structured expert elicitation with 600 experienced practitioners (contractors, consultants, project managers, and academics), we capture expert judgements on causal links between four technology clusters—AI-based progress detection, contract-aware BIM assistants, predictive cost–schedule twins, and AI-assisted design/constructability—and key project mechanisms and outcomes. All quantitative results in this study come from these expert judgements; no project-level before–after performance data are used. Accordingly, results are reported as pooled expert beliefs under stated scenarios, not empirically validated predictions. Hierarchical Bayesian models pool elicited probabilities into a Bayesian Network (BN) representing collective expert beliefs, rather than observed project performance. Monte Carlo simulations, Global Sensitivity Analysis (GSA), and Expected Value of Perfect Information (EVPI) are then used to explore technology-adoption and policy scenarios and to rank influential levers. Experts believe that, under idealized, high-readiness conditions, AI-enabled BIM Digital Twins could sharply reduce the probabilities of schedule delays >20% and cost overruns >10%. These figures are belief-based, optimistic upper-bound expectations derived from expert judgement, not empirically measured or validated impacts. The Bayesian Network therefore serves as an uncertainty-aware decision-support tool for early policy and investment decisions in data-poor contexts. It highlights the roles of training, organizational readiness, and client demand alongside technology adoption.

## Graphical abstract

**Figure:**
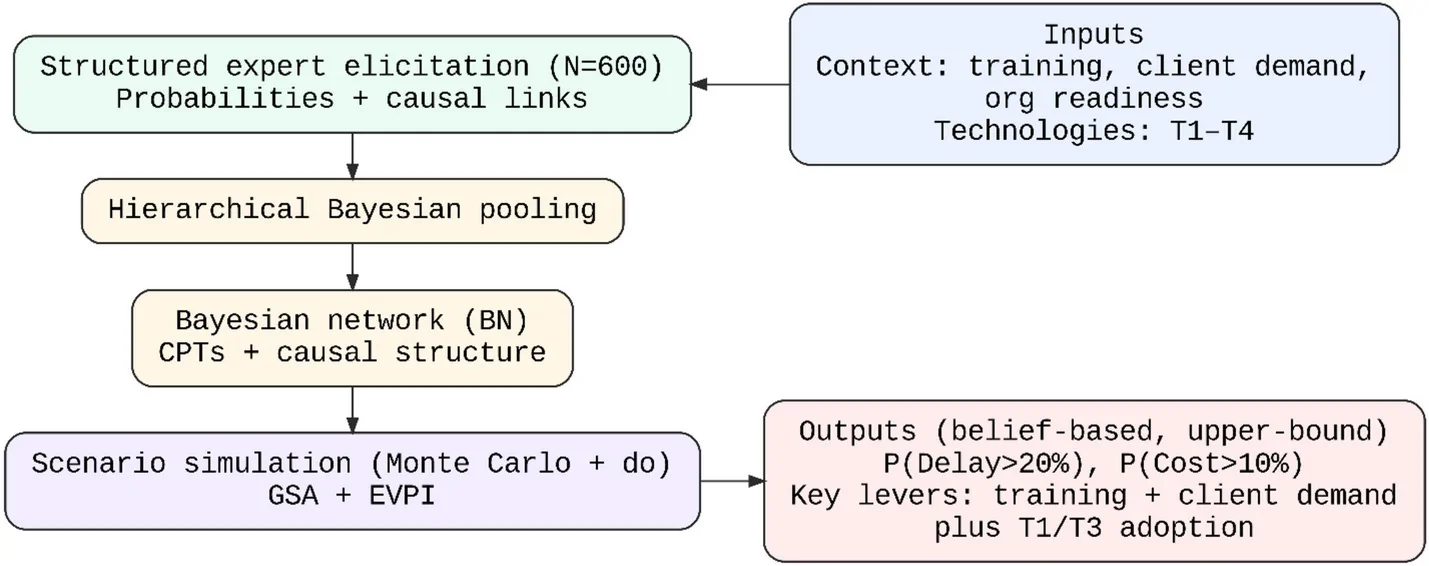
A schematic overview of the expert-elicited Bayesian Network and scenario analysis workflow is provided in the graphical abstract. Overview of the study: expert elicitation (*N* = 600) is pooled using hierarchical Bayesian models to parameterize a Bayesian Network (BN), which is then used for scenario simulation, sensitivity analysis, and Expected Value of Perfect Information (EVPI) to assess time–cost risks (delay > 20%; cost overrun > 10%).

## 1 Introduction

### 1.1 Background and problem statement

Jordanian building projects—especially of reinforced concrete residential and commercial buildings—are regularly plagued by significant delays in schedule and cost overruns. While Building Information Modelling (BIM) adoption in local design offices is growing, its use in practice is still mainly limited to three-dimensional (3D) modelling and clash detection. More advanced applications such as four−/five-dimensional (4D/5D) BIM, Artificial Intelligence (AI)-assisted progress tracking, and BIM-based Digital Twins are rarely integrated into day-to-day site management.

International studies have shown that BIM, AI, computer vision, and Digital Twins can significantly improve construction planning, monitoring, and control (Boje et al., 2020; Hsieh et al., 2026; Wang et al., 2024). However, most of this evidence comes from pilots and case studies in high-income contexts where, for example, client demand, regulatory frameworks and organizational capabilities vary significantly from those in countries such as Jordan. Local BIM research has been conducted on the level of adoption and barriers, but not yet concrete, quantifiable model that links specific AI/BIM Digital Twin technologies with the project’s time and cost by Jordanian institutional condition.

### 1.2 Aim and objectives

This paper fills that gap by developing an expert-elicited Bayesian Network (BN) that encodes how a large community of Jordanian experts believes AI-enabled BIM Digital Twins would influence cost-overrun and delay risks. The analysis is therefore entirely based on structured expert judgement, combined with hierarchical Bayesian pooling and Bayesian Network modelling, and not on before–after measurements from actual AI/BIM Digital Twin deployments. Accordingly, the Bayesian Network should be interpreted as a formalization of collective expert beliefs, rather than as an empirically validated predictive model. Throughout the paper, all numerical results are therefore reported as conditional expert-belief estimates under specified scenarios, rather than as out-of-sample predictive forecasts. Instead of assessing a few pilot projects, we systematically elicit and pool expert beliefs about causal relationships and probabilities and embed them in a transparent probabilistic model to facilitate uncertainty-aware scenario analysis and “what-if” policy exploration (Kabir et al., 2018; Pearl, 2009).

The objectives of the study are to (1) define a set of AI-enabled BIM Digital Twin technologies that are relevant to Jordanian building projects and identify key contextual, intermediate, and outcome variables and (2) elicit from 600 experts their beliefs about causal influences and probability distributions for delaying and cost overruns with and without these technologies (3) construct and parameterize a Bayesian Network that represent the aggregated expert judgements; and (4) use this model to explore technology-adoption and enabling-condition scenarios, to identify levers expected to yield the largest performance improvements.

### 1.3 Contributions and significance

The study makes three major contributions. First, it presents the first formal causal structure that connects AI/BIM Digital Twin technologies to project mechanisms and time–cost outcomes in Jordanian building construction. Second, it offers a quantified Bayesian Network, developed based on large-scale structured expert elicitation, appropriate for uncertainty-informed evaluation of hypothetical adoption scenarios. Third, it derives policy-relevant insights about the relative importance of technology, training and client/regulatory requirements for realizing the anticipated benefits of AI-enabled BIM Digital Twins.

All quantitative results in this study are based on structured expert judgement rather than observed before–after project data. The Bayesian Network therefore encodes collective expert beliefs under specified scenarios, not empirically validated predictive models. Throughout the paper, we interpret results as belief-based, upper-bound expectations conditional on idealised assumptions, rather than as forecasts of near-term performance.

### 1.4 Structure of the paper

The remainder of this paper is structured as follows. Section 2 reviews the literature on BIM, Digital Twins, AI in construction, and expert-elicited Bayesian Networks. Section 3 describes the belief-based research design, expert elicitation protocol, and Bayesian Network construction. Section 4 presents the simulated time–cost risk scenarios, sensitivity, and value-of-information results. Section 5 discusses the findings and their implications. Section 6 presents the conclusions, and Section 7 outlines limitations and directions for the future research.

## 2 Literature review

### 2.1 BIM, digital twins, and AI in building construction

Building Information Modelling (BIM) has evolved from a 3D design tool to an information-rich platform that includes 4D (time), 5D (cost), and broader lifecycle applications. Digital Twins are a further evolution of BIM, combining live or near-real-time data from the physical asset, which provides the capabilities of continuous monitoring, prediction and control (Boje et al., 2020; Wang et al., 2024). Recent conceptual and review work also argues that construction-oriented Digital Twins improve planning, progress tracking, and risk management and warrants attention to persistent challenges in data integration, semantic interoperability, and organizational change (Boje et al., 2020; Sacks et al., 2020).

Parallel advances in AI—particularly in computer vision and deep learning—enable progress to be detected automatically from images and point clouds (Hsieh et al., 2026), and natural language processing and large language models support interpretation of textual artefacts such as bills of quantities, contracts, and site reports. These capabilities enable integrated “AI-enabled BIM Digital Twin” configurations that couple sensing, analytics and rich information models. However, the literature is dominated by technology pilots, case studies, and proof-of-concept systems, with little quantified, causal assessment of impacts on schedule and cost performance (Eslahi et al., 2020; Wang et al., 2024).

### 2.2 BIM and digitalization in Jordan

Studies on Jordan and the broader MENA region show low to moderate levels of BIM adoption, and in the form of large design offices and specific flagship projects. BIM is mainly used for 3D modelling and clash detection, and there are limited 4D/5D applications which are manual. Commonly reported barriers include absence of client demand, absence of national BIM standards, high software and training costs, and interoperability issues, which are consistent with more general results of resistance to BIM innovations and construction digitalization (Tan et al., 2025).

Within Jordan, there is practically no documented evidence of the use of AI-augmented BIM and construction-oriented Digital Twins in regular building activities, and no formal models of the correlation between specific digital technologies and quantified risk bounded by time and cost under local institutional and market conditions. Recent efforts have started to merge BIM with machine learning together with metaheuristic optimization for predicting variation orders and overall project performance in terms of schedule and costs (e.g., Abuassi et al., 2025; Bassam and Al-Btoush, 2026; Al-Btoush et al., 2026; Al-Btoush et al., 2026). These studies, however, do not report an expert-elicited, causal Bayesian representation of AI-enabled BIM Digital Twins specific to the Jordanian building sector.

### 2.3 Expert elicitation and Bayesian Networks in construction

Expert elicitation is a recognized approach when empirical data are scarce, or technologies are emergent. Structured protocols can capture expert opinions about causal relationships and probabilities, which can then be encoded in Bayesian Networks (BNs). In construction and project management, BNs have been applied to safety, reliability, and cost/schedule risk analysis (Khodakarami and Abdi, 2014; Kabir et al., 2018), providing a transparent means to integrate diverse expert inputs, represent uncertainty, and perform scenario analysis.

In the more general reliability and risk literature, Kabir et al. (2018) identify BNs as especially well-suited to complex socio-technical systems for which the available data are incomplete and expert judgement is necessary. In construction itself, BN-based models have been employed to quantify the risk of construction project cost and to support decision-making under uncertainty, but again not yet as far as AI/BIM Digital Twin technologies applied to building projects in developing countries. Existing research on BIM and digitalization in Jordan mainly covers levels of adoption, barriers and perceived benefits, yet international research on application of AI, computer vision and Digital Twins is dominated by pilots and case studies (Boje et al., 2020; Eslahi et al., 2020; Wang et al., 2024). Quantified, causal impact assessments of the explicit connections between particular AI/BIM Digital Twin technologies and time and cost risks in building projects in developing countries are virtually non-existent. Moreover, to our knowledge, large-scale expert elicitation, hierarchical Bayesian inference and scenario-based BN modelling have never been combined before to describe anticipated effects of AI-enabled BIM Digital Twins in the Jordanian building sector. This research helps to fill that void. Table 1 summarizes selected BN and Digital Twin studies and highlights how the present work differs by focusing on AI-enabled BIM Digital Twins, using large-scale expert elicitation, and modelling time–cost risks in Jordanian building projects.

**Table 1:** Summary of selected related studies and the gap addressed by this study.

Study	Context/focus	Method/data	Gap relative to this study
Khodakarami and Abdi (2014)	Project cost risk in construction	Bayesian Network; expert and project data	No AI/BIM Digital Twins; not focused on developing-country context
Kabir et al. (2018)	Reliability, risk, and maintenance (review)	Review of BN applications	No construction-specific Digital Twin or AI/BIM focus
Boje et al. (2020)	Construction Digital Twin (conceptual)	Conceptual framework; literature review	No quantified time–cost risk model; no expert-elicited BN
Sacks et al. (2020)	Digital Twin information systems	Conceptual and case-based	No formal probabilistic model of schedule/cost risk
Wang et al. (2024)	Digital Twins in construction project management	Systematic review	No causal BN; limited attention to developing countries
Abuassi et al. (2025)	Jordanian construction project performance	Machine learning + metaheuristics; project data	No expert-elicited BN; no explicit AI/BIM Digital Twin adoption
Bassam and Al-Btoush (2026)	Variation orders in Jordanian projects	BIM + ML + optimization; project data	No explicit Digital Twin focus; no BN of expert beliefs
This study	Jordanian building project time–cost risks under AI-enabled BIM Digital Twins	Expert elicitation + hierarchical Bayesian models + BN; simulations, GSA, EVPI	First causal BN of expert beliefs on AI/BIM Digital Twins in Jordanian building projects

GSA, global sensitivity analysis; EVPI, Expected Value of Perfect Information; ML, Machine Learning.

## 3 Methodology

### 3.1 Research design overview

The analysis is entirely belief-based: probabilities and causal links are elicited from experts and then aggregated using hierarchical Bayesian models and a Bayesian Network. No project-level before–after data on AI/BIM Digital Twin deployments are used.

**Figure 1:**
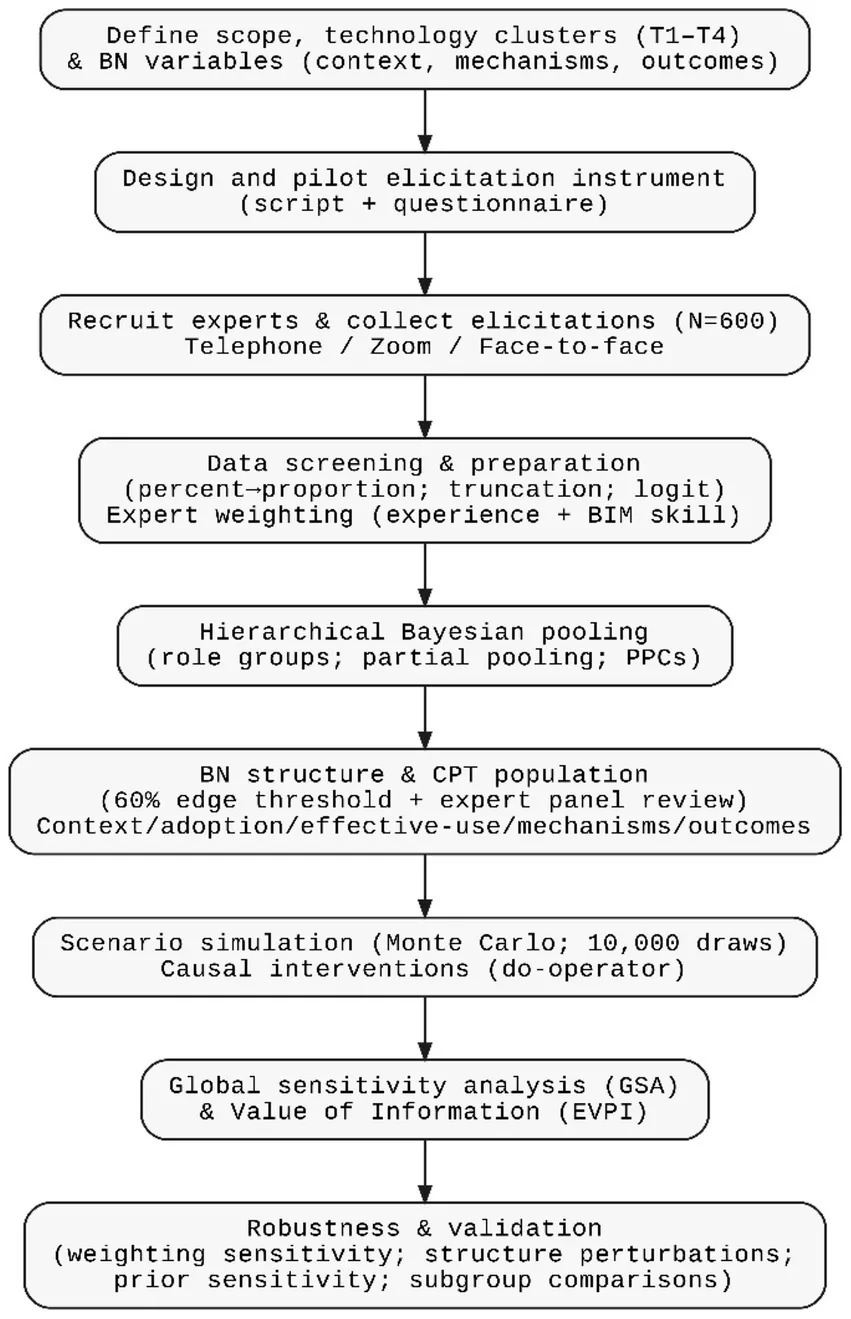
Overview of the research process, from expert elicitation through hierarchical Bayesian modelling to BN-based scenario analysis.

This study combines structured expert elicitation with hierarchical Bayesian inference (Gelman et al., 2013) and Bayesian Network (BN) modelling (Kabir et al., 2018) to evaluate how AI-enabled BIM Digital Twin technologies may affect schedule and cost outcomes in Jordanian building projects. A schematic overview of the research process, from expert elicitation through hierarchical Bayesian modelling to BN-based scenario analysis, is presented in Figure 1.

Between February 2022 and February 2026, we invited 1,000 construction-sector experts and obtained 600 complete elicitations (60% response). Data were collected via telephone (330), Zoom (270), and face-to-face (100) interviews. Respondents identified a single dominant professional role (contractor, consultant, project manager, or academic), used for subgroup analyses and hierarchical pooling.

Participation involved non-sensitive professional opinions; informed consent was obtained, and all responses were anonymized before analysis.

All responses were checked for completeness and internal consistency; records that were not informative on all core probability items were disqualified. Percentages were transformed to proportions in [0,1] and truncated to [1e-6, 1 − 1e-6] before being transformed into logit. Categorical covariates were always encoded. The continuous expert weight calculated per respondent was the sum of the normalized experience and the rescaled self-rated BIM skill (Section 3.2); these weights were used as likelihood weights in the hierarchical models to give more weight to more experienced and more BIM-competent experts without dropping any observations. Since the sample was based on an outreach pool and was obtained through mixed modes across 4 years, there might be selection and mode effects; we thus present subgroup findings and sensitivity and robustness analyses (Section 3.7).

In addition, the use of professional networks and snowball sampling may have led to selection bias, for example over-representing practitioners who are more interested in digital technologies. This could make the perceived benefits of AI-enabled BIM Digital Twins somewhat more optimistic than in the full practitioner population.

Recruitment and sampling: Potential respondents were located via professional contacts, engineering and contractors’ associations in Jordan, alumni list of the universities and training programs, and referrals among the first contacts (snowball sampling). E-mails and telephone invitations were issued to those with at least 5 years’ experience in building projects and some familiarity with BIM or digital construction tools. The aim of the sampling was not to obtain a statistically representative sample of all Jordanian practitioners, but rather to achieve a broad cross-section of experienced experts (contractors, consultants, project managers, and academics) who could offer informed opinions on AI-enabled BIM Digital Twin technologies.

#### 3.1.1 Data and materials availability

Anonymized aggregate summaries and BN parameter tables are provided in the appendices. De-identified row-level elicitation data and example analysis scripts (PyMC3/plotting) can be shared with qualified researchers on reasonable request, subject to a data-use agreement to protect respondent confidentiality.

#### 3.1.2 Elicitation protocol and quality control

An elicitation interview that was structured, directed by researchers and carried out by telephone, Zoom, and face-to-face was conducted using a standardized script and questionnaire tested on five elderly practitioners. The instrument consisted of short samples of the training on reporting the probability, basic probability questions to serve as a baseline and a risk on technology and delay and costs, Likert-scale causal-link questions, and metadata of the respondents. Interviewers provided numeric examples, clarified scenario assumptions, and recorded responses.

Data quality was managed through several steps. First, screening of responses was done in the field to check completeness and apparent inconsistencies, and clarification was immediately requested. Second, in the analysis stage, probabilities were truncated to [1e-6, 1 − 1e-6] to prevent numerical instability in the logit transformation, and cases with non-responded core items were excluded. Third, internal quality and sensitivity analyses were conducted, including comparing the medians of core elicited probabilities between interview modes (telephone/Zoom/face-to-face), screening out missing, implausible, or internally inconsistent responses and recording their treatment, and confirming that posteriors based on pooling remained similar under alternative weighting schemes (weighted and equal weight). Appendix C shows the interviewer script and selected instrument items.

### 3.2 Data preparation and expert weighting

The elicitation tool was piloted in advance on five senior practitioners before the full-scale survey was conducted. The probability-elicitation items were refined using pilot feedback, which mainly added more understandable text and numerical formats for describing the beliefs as percentages in the probability that they would appear at baseline and technology-conditional delay and cost risks. The last questionnaire was introduced with short guidelines on how to interpret the scenarios and make probability judgements to minimize the possibility of misunderstanding and anchoring on round numbers.

All survey responses were checked for completeness and basic consistency. Records that had no value of key probability items (baseline and technology-conditional delay and cost risks) were excluded. Percentages were transformed to [0,1] proportions and truncated to [10^−6^, 1–10^−6^] to prevent numerical problems in the logit transformation. Ordinal encodings were performed to encode categorical variables (e.g., training level, demand by clients, and BIM-use frequency), which were to be subjected to further analysis based on the provided ordinal encodings.

Each respondent reported a single dominant professional role (contractor, consultant, project manager, and academic). This dominant role was used to assign experts to four mutually exclusive groups g ∈ {contractor, consultant, PM, academic} for the hierarchical Bayesian models in Section 3.3. In the descriptive statistics (Appendix A, Table A1), respondents could indicate experience in multiple roles, so role percentages exceed 100%; for modelling, however, each observation enters exactly one group.

To reflect variation in expected reliability and relevance of judgements, we derived a continuous weight w_i for each respondent i, combining years of experience and self-rated BIM skill. Experience categories (<5, 5–10, 11–20, >20 years) were mapped to mid-points (2.5, 7.5, 15, 25 years) and linearly normalized to E_i ∈ [0,1]. Self-rated BIM skill on a 1–5 Likert scale was rescaled to B_i ∈ [0,1] via B_i = (skill_i − 1)/4. The raw weight was w_i = 0.5E_i + 0.5B_i, and normalized to mean 1 across *N* = 600 experts is expressed as.


w_i∗=w_i/[(1/N)∑_{j=1}^Nw_j]

These normalized weights entered the hierarchical models as likelihood weights, so that more experienced and more BIM-proficient experts had proportionally greater influence while retaining all responses in the analysis.

Weighting rationale and sensitivity. The weighting scheme was chosen to reflect the expectation that experts with more experience and higher BIM proficiency provide more reliable judgements, while still retaining all responses. To assess robustness, we re-estimated the key pooled probabilities and scenario outcomes using alternative schemes (equal weights, experience-only weights, BIM-skill-only weights). The main qualitative conclusions regarding baseline risks, relative risk reductions, and the ranking of influential factors were unchanged; numerical results for all schemes are reported in Appendix A (Table A6).

### 3.3 Hierarchical Bayesian parameterization of CPTs

For each elicited probability p_i reported by expert i (converted from percentage to a proportion and clipped to [*ε*, 1 − ε] with ε = 10^−6^), we define the logit transform as follows:


y_i=logit(p_i)=log[p_i/(1−p_i)]

Experts are assigned to one of four mutually exclusive role groups g ∈ {contractor, consultant, PM, academic}, with g(i) denoting the group of expert i. Each respondent has a normalized continuous weight w_i* (Section 3.2) reflecting experience and self-rated BIM skill.

Observation model (individual level):


y_i∼Normal(μ_{g(i)},σ_within),i=1,…,N

Group level (partial pooling):

μ_g ∼ Normal(μ_pop, σ_between), for each group g.

Hyperpriors (weakly informative):

μ_pop ∼ Normal(0, 2),

σ_between ∼ HalfNormal(1),

σ_within ∼ HalfNormal(1).

Weighting implementation: normalized expert weights w_i* are incorporated as likelihood weights by scaling each observation’s log-likelihood as follows:


logL=∑{i=1}^Nw_i∗·logNormal(y_i∣,μ{g(i)}∣,σ_within)

The qualitative results on baseline risks, relative risk reduction, and the ranking of influential factors did not change significantly, and numerical results are given for all schemes in Appendix A (Table A6).

Each of the four core conditional probabilities required for the BN—P(Delay>20% | baseline), P(Delay>20% | T1 adopted), P(Cost>10% | baseline), P(Cost>10% | T3 adopted)—was modelled separately. The thresholds of delay >20% and cost overrun >10% were chosen in consultation with senior practitioners as representative cut-offs for “substantial” schedule and budget overruns in typical Jordanian reinforced-concrete building projects and are consistent with values used in previous construction risk studies. Models were fitted with Hamiltonian Monte Carlo (PyMC3 version 3.11.5): four chains, 1,000 warm-up iterations, and 2,000 post-warm-up draws per chain, targeting an accept rate of 0.95. Convergence was assessed using Rˆ (target ≈ 1.00) and effective sample size (ESS); posterior predictive checks (PPCs) compared simulated expert reports to observed responses (Gelman et al., 2013). Posterior medians of group- and population-level probabilities were obtained by inverse-logit transformation of μ_g and μ_pop and used to populate BN Conditional Probability Table (CPT) entries; 90 and 95% credible intervals (CIs) quantify uncertainty.

### 3.4 Constructing the Bayesian Network and CPT population

**Figure 2:**
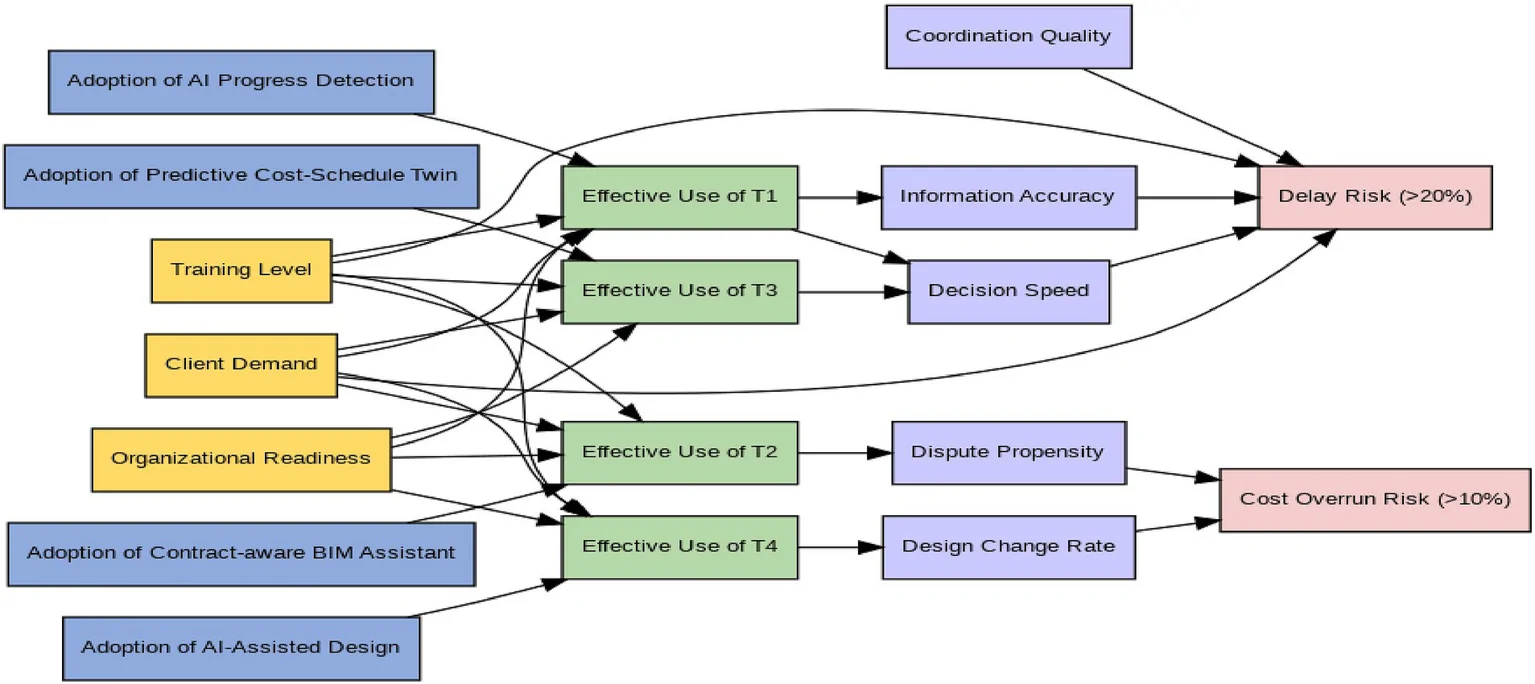
Bayesian network structure for AI-enabled BIM digital twin technologies, contextual enablers, intermediate mechanisms, and schedule/cost risk outcomes in Jordanian building projects. The columns (from left to right) group context nodes (Training_Level, Client_Demand, and Org_Readiness), technology-adoption nodes (Adoption_T1–Adoption_T4), effective-use nodes (EffectiveUse_T1–EffectiveUse_T4), intermediate mechanism nodes (Info_Accuracy, Decision_Speed, Coordination_Quality, Design_Change_Rate, and Dispute_Propensity), and outcome nodes (Delay_Risk and Cost_Risk). Directed edges represent expert-elicited causal influences, with T1–T4 ordered consistently from top to bottom. The arrows follow the assumed causal order “context → adoption/effective use → mechanisms → outcomes,” as described in Section 3.4.

The Bayesian Network (BN) framework was created to represent how experts assume that Digital Twin technologies in Jordanian building projects, together with contextual enablers and intermediate mechanisms, jointly affect time and cost performance (Khodakarami and Abdi, 2014; Kabir et al., 2018). The dependency chain is: context (Training_Level, Client_Demand, Org_Readiness) → adoption/effective use (T1–T4) → intermediate mechanisms → outcomes (Delay_Risk, Cost_Risk). In simple terms, context affects which technologies are adopted and used effectively; these technologies then change intermediate mechanisms on projects, and those mechanisms, together with context, determine delay and cost risks. CPTs are populated using (i) elicitation-sample frequencies for priors/baselines, (ii) hierarchically pooled expert probabilities for baseline vs. technology-conditional outcome risks, and (iii) simple transparent rule-based mappings for adoption/effective use and Likert-to-probability mechanism shifts. Key modelling choices (edge-inclusion threshold, Likert-to-probability mapping, and context-effect bounds) were selected for transparency and tested for robustness as described below. The first qualitative structure was obtained from the answers provided in Section B of the elicitation tool, with experts pointing out direct positive or negative effects between variable pairs. Where over 60% of respondents said they were directly influenced, a directed edge was added to strike a balance between inclusiveness and strength. This threshold was chosen to represent a clear majority view while avoiding an overly dense network; exploratory checks with alternative thresholds of 50 and 70% produced similar qualitative conclusions about key dependencies. We chose this simple rule instead of data-driven structure learning to keep the network transparent and closely aligned with the majority expert view. The draft network was then reviewed by a panel of five senior experts to ensure conceptual coherence, alignment, and absence of cycles, resulting in only minor adjustments. Figure 2 shows the final BN structure, including the main context, technology-adoption, effective-use, intermediate-mechanism, and outcome nodes and their directed dependencies. In this structure, context nodes (training, client demand, and organizational readiness) affect technology-adoption and effective use; these in turn influence intermediate mechanisms (information accuracy, decision speed, coordination, design changes, and disputes), and these mechanisms collectively affect the delay and cost-risk outcome nodes.

The final BN represents experts’ collective causal model and comprises the following:

Context nodes (three states): Training_Level, Client_Demand, and Org_Readiness ∈ {Low, Medium, High}

Technology-adoption nodes (binary): Adoption_T1–Adoption_T4 ∈ {Off, On}, indicating adoption of the four AI/BIM Digital Twin clusters.

Effective-use nodes (binary): EffectiveUse_T1–EffectiveUse_T4 ∈ {Ineffective, Effective}, indicating whether an adopted technology is used effectively.

Intermediate mechanism nodes (three states): Info_Accuracy, Decision_Speed, Coordination_Quality, Design_Change_Rate, and Dispute_Propensity ∈ {Low, Medium, High}.

Outcome nodes: Delay_Risk (probability that delay >20%) and Cost_Risk (probability that cost overrun >10%), each conditioned on a scenario node (baseline vs. technology adopted) and on the three context nodes (Training_Level, Client_Demand, and Org_Readiness), with intermediate mechanisms influencing them through the CPT construction described below.

#### 3.4.1 CPTs for context, adoption, and effective use

Context node priors were set to the empirical distributions observed in the survey. Adoption_T1–T4 CPTs were specified conditional on context to reflect experts’ aggregated views on how training, client demand and organizational readiness affect adoption likelihood. EffectiveUse nodes are probabilistic functions of Adoption and context. Simple rule-based CPTs, calibrated to elicited Likert assessments, were specified, for example: if Adoption_T1 = Off then P(EffectiveUse_T1 = Effective) ≈ 0; if Adoption_T1 = On and Training_Level and Org_Readiness are High then P(EffectiveUse_T1 = Effective) is high; intermediate configurations map to intermediate probabilities. These CPTs encode the consensus that adoption alone is insufficient—effective use requires enabling conditions.

#### 3.4.2 CPTs for intermediate mechanism nodes

Each intermediate node is a child of the relevant EffectiveUse_T* node(s) and, where appropriate, selected context nodes. Baseline distributions over {Low, Medium, High} were derived from empirical frequencies. For each technology–mechanism pair, mean 1–5 Likert impact scores were linearly rescaled to an impact weight w ∈ [0,1] via w = (s − 1)/4. This linear mapping was adopted as a simple, transparent transformation that preserves the relative strength of perceived impacts without over-parameterizing the CPTs. We preferred this direct linear rescaling over more complex latent-variable models because it is easy to interpret and adequate for the level of detail and data volume in this study. Trial runs with slightly different monotonic mappings (e.g., mild nonlinear rescaling) did not change the relative ranking of influential mechanisms or the main scenario conclusions.

Under EffectiveUse = Effective, a fraction w of mass is shifted from Low and Medium to High:


$$\begin{array}{l} p\_H^{’}=p\_H+w\cdot (p\_L+p\_M),p\_L^{’}=\\ p\_L\cdot (1-w),p\_M^{’}=p\_M\cdot (1-w) \end{array}$$

followed by renormalization. Under Ineffective use, the baseline distribution is retained. This transparent mapping converts ordinal impact judgements into probabilistic shifts toward favourable mechanism states.

#### 3.4.3 CPTs for outcome nodes (Delay_Risk and Cost_Risk)

Delay_Risk is conditioned on a scenario node (baseline vs. T1 adopted) and on Training_Level, Client_Demand and Org_Readiness. Cost_Risk is analogously conditioned on a scenario node (baseline vs. T3 adopted) and the same contextual enablers. The hierarchical Bayesian models (Section 3.3) were fitted separately for P(Delay>20% | baseline), P(Delay>20% | T1 adopted), P(Cost>10% | baseline), and P(Cost>10% | T3 adopted). Posterior draws from these models provide base adverse-outcome probabilities p_base used in outcome CPTs; posterior medians (inverse-logit of μ_g and μ_pop) populate primary CPT entries, and posterior samples are used in simulation to propagate parameter uncertainty.

#### 3.4.4 Contextual modulation of outcome probabilities

Context nodes modulate p_base multiplicatively. For each context variable C ∈ {Training, Client, Org}, factors f_C(state) were derived from expert judgements, with Medium normalized to 1.0 and Low/High set within [0.7, 1.3] to remain consistent with elicited ranges. The range [0.7, 1.3] was selected to represent moderate context effects consistent with elicited responses (Table A4) and to avoid extreme adjustments that would dominate the elicited baseline probabilities; exploratory tests with wider ranges (e.g., 0.6–1.4) produced very similar rankings of scenarios and influential variables. This parsimonious multiplicative scheme was chosen to keep CPTs interpretable while still reflecting the direction and approximate magnitude of contextual influences reported by experts. The adjusted adverse-outcome probability for a given context configuration is,


p_adj=clamp(p_base×f_Training×f_Client×f_Org,0,1)

with the complementary state assigned 1 − p_adj. This parsimonious scheme captures how contextual enablers amplify or attenuate base risks while keeping CPTs interpretable.

#### 3.4.5 Linking mechanisms and core risk estimates

Intermediate mechanism nodes make the perceived causal pathways explicit (information accuracy, decision speed, coordination, design-change rate, dispute propensity), while the scenario analyses anchor core outcome probabilities to the hierarchically modelled expert posteriors p_base. Thus, intermediate nodes represent mechanisms by which technologies and context affect outcomes, whereas outcome CPTs remain firmly grounded in the formally elicited and pooled probability estimates. Mechanism-node CPTs are obtained by shifting baseline {Low, Medium, High} distributions using rescaled Likert impact weights, while outcome-node CPTs (Delay_Risk, Cost_Risk) start from the pooled expert probabilities. They are then moderately adjusted by the context modulation factors.

This way, the CPTs directly implement the idea that context → adoption/effective use → mechanisms → delay and cost outcomes.

### 3.5 Scenario simulation, causal interventions, and uncertainty propagation

To explore the probabilistic consequences of different technology-adoption and policy settings, we used Monte Carlo simulation on the BN, directly leveraging posterior uncertainty in CPT parameters. This allows uncertainty in expert judgements to be fully propagated to the outcome probabilities.

For each of 10,000 Monte Carlo draws per scenario, we performed:

CPT parameter sampling. For outcome nodes (Delay_Risk and Cost_Risk), CPT entries based on p_base were obtained by sampling from the posterior distributions of the hierarchical models in Section 3.3. For intermediate nodes derived from Likert-based impacts, CPT rows were sampled using the baseline distributions and impact-weight mapping described in Section 3.4, reflecting uncertainty in both baseline states and perceived impacts.

BN instantiation: A BN instance was constructed for that draw, populated with the sampled CPTs for all nodes.

Causal interventions (do-operator): Causal interventions were applied by setting the states of selected nodes [e.g., do(Adoption_T1 = On); do(Training_Level = High)], which cuts incoming edges to those nodes for that draw, following Pearl’s do-calculus semantics (Pearl, 2009). This allows us to simulate the effects of forcing adoption or enabling conditions, rather than merely conditioning on observations.

Inference and outcome recording: Exact or approximate BN inference was then used to compute the posterior predictive probabilities of Delay_Risk and Cost_Risk under the specified intervention. These probabilities were stored for that draw.

This procedure yields 10,000 samples of P(Delay>20%) and P(Cost>10%) for each scenario. We summarize the resulting posterior predictive distributions by their medians and 90% credible intervals, providing uncertainty-aware estimates of expected project performance under contrasting technology-adoption and policy configurations.

### 3.6 Sensitivity analysis and value of information (VoI)

To identify the most influential causes of delay and cost risks, and priorities where more information would be most valuable, we performed Global Sensitivity Analysis (GSA) and approximated Expected Value of Perfect Information (EVPI) using the Monte Carlo outputs.

For GSA, we quantified the contribution of each input node (technology-adoption variables, context factors, intermediate mechanisms) to the variance of the Delay_Risk and Cost_Risk. A variance-based decomposition, conceptually similar to Sobol total effect indices, for the simulated outcome probabilities across random draws of CPT parameters and node state was directly used on simulated data (Kabir et al., 2018). For each input, its total-effect index reflects its overall contribution as well as its interaction with other variables; the higher its index, the more leverage it has from interventions or uncertainty reduction.

For EVPI, we approximated the marginal value of knowing the results of subsampling the learning nodes perfectly (e.g., Training_Level, Client_Demand, Adoption_T1, and Adoption_T3). For each target variable and possible state of the variable, we conditioned the BN on that state and recomputed the distribution of Delay_Risk and Cost_Risk for several Monte Carlo draws. The EVPI for a variable was then calculated as the expected absolute decrease in the probability of an adverse outcome compared to the unconditioned scenario if the true state of the variable were known before decisions were taken (Pearl, 2009; Kabir et al., 2018). These analyses provide support for ranking of variables by their influence and information value, supporting priorities for policy, data collection and capacity building.

### 3.7 Model validation and robustness checks

We used several methods to evaluate the validity and robustness of the model (Gelman et al., 2013; Kabir et al., 2018). First, for face validation, a panel of five senior construction experts (two contractors, two consultants, and one academic) who did not participate in the initial elicitation reviewed the final BN structure (Figure 1) and key CPTs. They were confident that the directions of influence and sizes of probabilities were conceptually consistent with domain knowledge and plausible regarding Jordanian building projects.

Second, posterior predictive checks (PPCs) were used to assess the fit of the hierarchical Bayesian models and the BN. For each of the 4 core conditional probabilities, we produced posterior predictive samples of individual expert reports and compared their distribution with the observed responses. The PPCs suggested that the models reproduce the central tendencies and dispersion of elicited probabilities without systematic misfit.

Third, robustness analyses were conducted. We tested alternative schemes to weight expert opinions (this included equal weighting) and found that qualitative conclusions about influential factors and risk reductions did not change. We tested perturbations to the BN structure by adding and removing a small number of weakly supported edges and repeated simulations; the ranking of influential factors and relative effects of adoption scenarios were stable. Finally, we evaluated sensitivity to prior choices by slightly modifying the scales of hyperpriors (μ_pop, σ_between, and σ_within); posterior CPTs and scenario outcomes were relatively insensitive to choices of weakly informative priors, suggesting that sufficient information from the data was available for the weakly informative priors selected.

## 4 Bayesian network construction

### 4.1 Posterior summaries for pooled probabilities

The hierarchical Bayesian analysis yielded pooled estimates of key time- and cost-risk probabilities, disaggregated by expert role and accounting for within- and between-group variability (Gelman et al., 2013). These estimates summarize experts’ collective beliefs about current practice and idealized technology-adoption scenarios, rather than empirically observed outcomes.

For schedule risk under current practice, experts perceive a very high likelihood of substantial delays. The posterior median for the population-level probability P(Delay > 20% | baseline) is 0.78 (95% CI [0.75, 0.81]). By role, contractors report the highest median (0.81, 95% CI [0.76, 0.86]), academics the lowest (0.76, 95% CI [0.70, 0.81]), with consultants and project managers in between (0.77 and 0.79). This indicates broad consensus that significant schedule overruns are typical in Jordanian building projects.

Under an explicitly idealized scenario in which T1 (AI-based progress detection) is widely and effectively adopted, experts anticipate a marked reduction in schedule risk. These reductions are computed directly from the elicited baseline and technology-conditional probabilities and their hierarchical Bayesian pooled posteriors (Section 3.3), not from observed project performance data. The posterior median P(Delay > 20% | T1 adopted) falls to 0.09 (95% CI [0.06, 0.13]). These values represent an optimistic upper bound under idealized enabling conditions and should not be interpreted as near-term realized effects. Contractors’ median falls to 0.10 (95% CI [0.04, 0.20]) and academics’ median falls to 0.07 (95% CI [0.02, 0.15]). These values represent upper-bound expectations under mature implementation and strong enablers (high training, organizational readiness, client support), not short-term effects of current pilots.

For cost risk, the pooled baseline probability P(Cost > 10% | baseline) is 0.26 (95% CI [0.23, 0.29]). Contractors again report the highest median (0.29, 95% CI [0.23, 0.35]) and academics the lowest (0.24, 95% CI [0.19, 0.30]), with other roles in between, reinforcing the perception that cost overruns are common. Under an idealized scenario of widespread, effective adoption of T3 (predictive cost–schedule Digital Twin), the median P(Cost > 10% | T3 adopted) drops to 0.04 (95% CI [0.03, 0.06]); contractors’ median is 0.05 (95% CI [0.02, 0.11]) and academics’ 0.03 (95% CI [0.01, 0.08]). These are belief-based upper bounds consistent with emerging Digital Twin evidence (Boje et al., 2020; Wang et al., 2024), not effects estimated from before–after project data.

In other words, these scenario results show what experts think could be achievable under mature, well-enabled adoption. They should be read as upper-bound belief scenarios, not as short-term empirical impact estimates for current Jordanian projects.

### 4.2 Scenario forecasts (Monte Carlo simulations)

Monte Carlo simulations (10,000 draws on each scenario) with the BN and posterior CPTs described in Section 3 were carried out as scenario-based simulations. This allows comparison of schedule and cost risks across technology implementations and conditions within the entire context, when uncertainty is fully propagated (Khodakarami and Abdi, 2014; Kabir et al., 2018). The scenarios are completely variable between present practice and the utopian case of maximum adoption and are based on the belief of experts aggregated instead of the actual implementations.

Illustrative scenarios include the following:

Baseline (no technology intervention).

P(Delay > 20%) ≈ 0.785 (90% CI [0.72, 0.84]);

P(Cost > 10%) ≈ 0.265 (90% CI [0.21, 0.32]).

Moderate adoption (T1 and T3 at 30% adoption).

P(Delay > 20%) ≈ 0.420 (90% CI [0.32, 0.53]);

P(Cost > 10%) ≈ 0.120 (90% CI [0.07, 0.19]).

Idealized high adoption with strong enablers (T1 + T2 + T3 On; high training, organizational readiness, and client demand).

P(Delay > 20%) ≈ 0.075 (90% CI [0.03, 0.15]);

P(Cost > 10%) ≈ 0.038 (90% CI [0.02, 0.07]).

Adoption without readiness (T1 + T3 On; low training; medium client demand).

P(Delay > 20%) ≈ 0.360 (90% CI [0.25, 0.50]);

P(Cost > 10%) ≈ 0.140 (90% CI [0.09, 0.23]).

These very low probabilities in the “idealized high adoption with strong enablers” case should be understood as optimistic, belief-based upper-bound estimates. They reflect what experts think could be achievable in the long term under widespread, effective adoption of AI/BIM Digital Twins combined with high training, strong organizational readiness, and clear client demand. They are not forecasts of near-term improvements under current Jordanian conditions and should not be interpreted as typical or guaranteed impacts.

The idealized high-adoption scenario should be interpreted as an optimistic upper-bound belief case under mature implementations and strong socio-technical enablers, rather than a short-term prediction for current pilot deployments.

**Figure 3:**
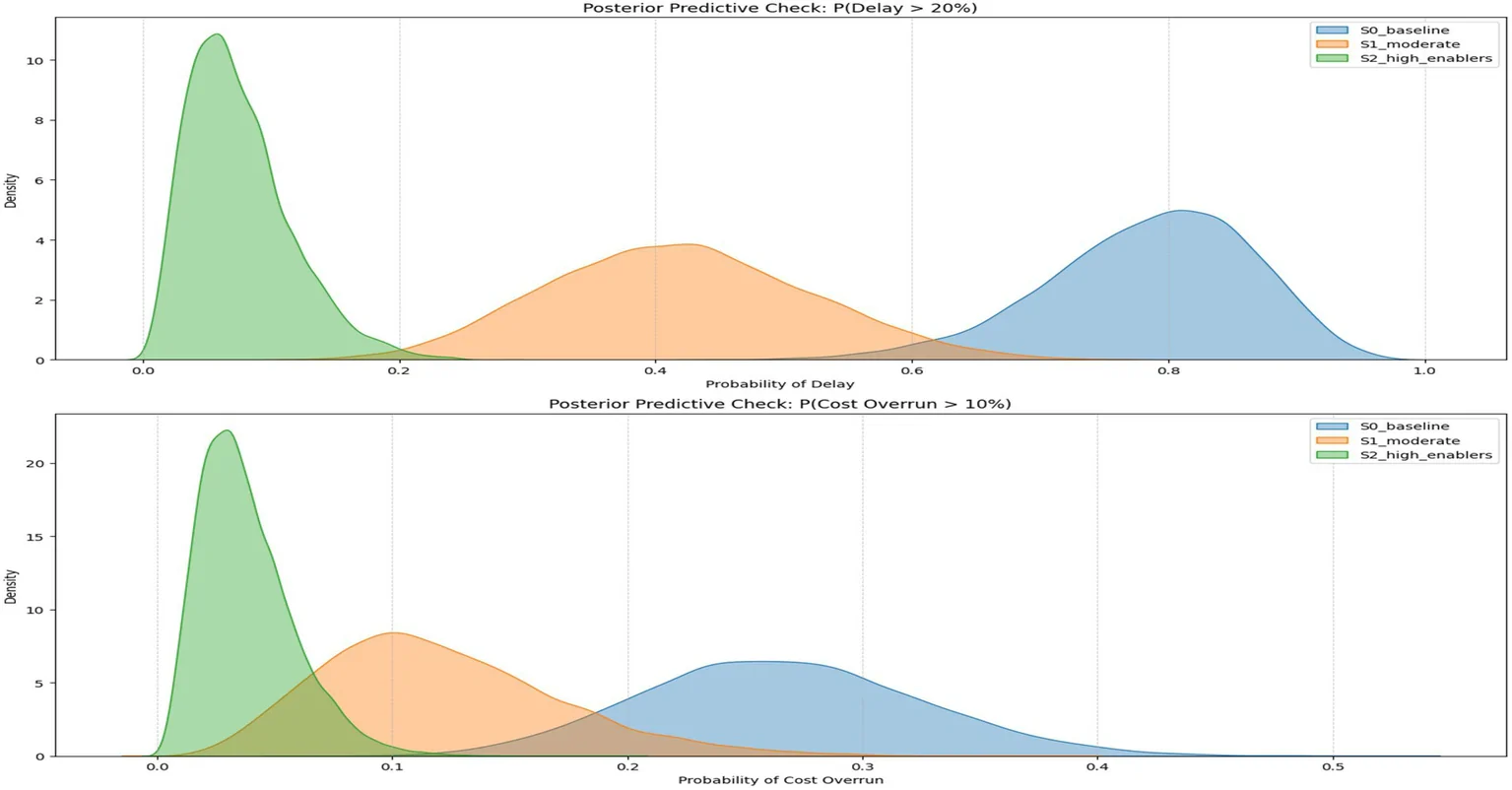
Posterior predictive distributions for P(Delay > 20%) and P(Cost > 10%) under selected technology-adoption scenarios (baseline, moderate adoption, idealized high adoption with strong enablers, adoption without readiness). Solid curves show simulated distributions; dashed vertical lines indicate elicited expert mean probabilities for each scenario. T1 and T3 denote AI-based progress detection and the predictive cost–schedule Digital Twin, respectively (Section 3.4).

Figure 3 presents posterior predictive distributions for P(Delay > 20%) and P(Cost > 10%) across these scenarios, highlighting both central tendencies and uncertainty ranges and comparing simulated outcomes with elicited expert means.

### 4.3 Sensitivity analysis

The output of the Monte Carlo simulation was subjected to Global Sensitivity Analysis (GSA) to measure the contribution of each input node to the range of variation of the Delay_Risk and Cost_Risk (Kabir et al., 2018). Using a variance-based decomposition analogous to Sobol total-effect indices, we approximated the total effect of each input on the output (interactions with other variables).

For P(Delay > 20%), the largest total-effect indices were associated with Training_Level (0.34), Adoption_T1 (AI-based progress detection; 0.28), and Client_Demand (0.22). This indicates that training and client-driven mandates are at least as critical as the mere presence of T1 for reducing delay risk, consistent with socio-technical perspectives on BIM and digitalization (Chong et al., 2016; Tan et al., 2025).

For P(Cost > 10%), the dominant drivers were Adoption_T3 (predictive cost–schedule Digital Twin; 0.31), Training_Level (0.27), and Client_Demand (0.20). While T3 is perceived as highly effective for cost control, its impact is strongly moderated by human-capital and client-requirement factors.

**Figure 4:**
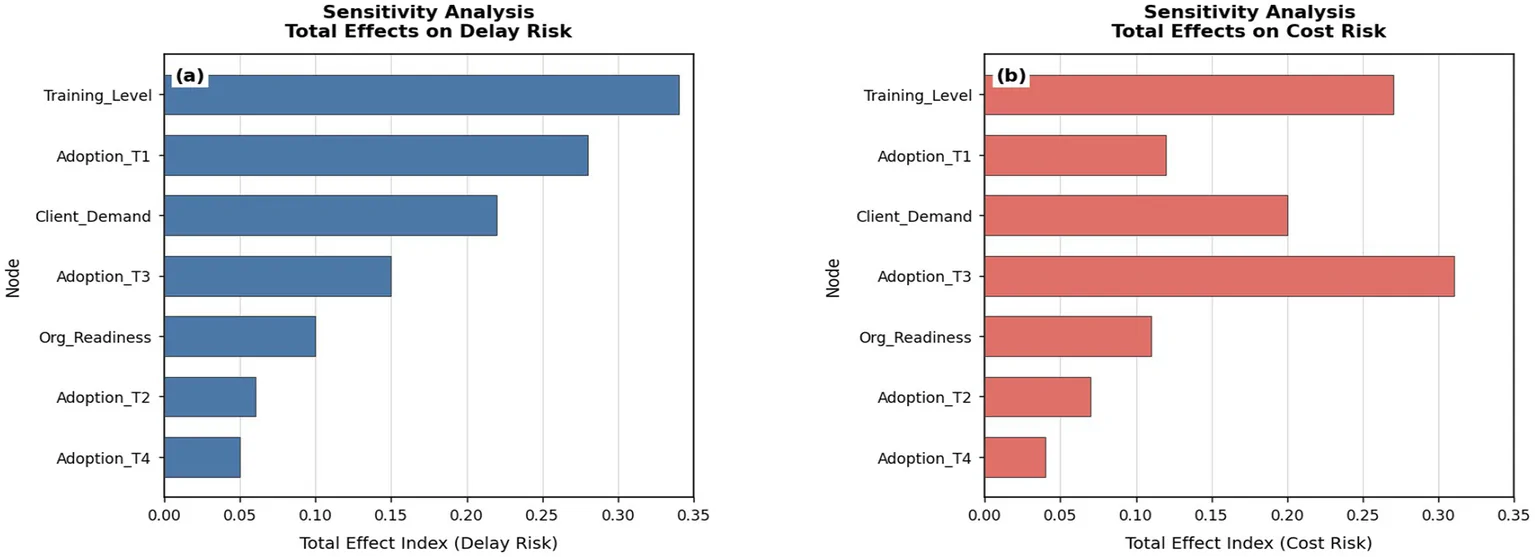
Total-effect sensitivity indices for the Bayesian Network. The left panel shows the contribution of each input node to the variance of the probability of Delay > 20%, and the right panel shows the corresponding contributions for the probability of Cost overrun > 10%. Bars show the contribution of each input (context, technology-adoption, and selected intermediate mechanisms) to the variance of the corresponding outcome, including interaction effects.

Figure 4 shows the ranked total-effect indices for key inputs, separately for delay and cost outcomes, highlighting the relative leverage of training, technology-adoption, and client demand.

Higher total-effect indices indicate greater overall influence (including interactions) on outcome variance.

### 4.4 Value of information (VoI) analysis

To further prioritise interventions and data-collection efforts, we estimated the Expected Value of Perfect Information (EVPI) for selected input nodes using the BN simulations (Pearl, 2009; Kabir et al., 2018). For each target variable and each of its possible states, we conditioned the BN on that state, recomputed the distributions of P(Delay > 20%) and P(Cost > 10%), and compared them with the unconditioned case. The EVPI was approximated as the expected absolute reduction in adverse-outcome probability if that variable were known with certainty before decisions are made.

For P(Delay > 20%), resolving uncertainty about Training_Level yields the largest EVPI (≈0.12 absolute reduction), followed by Client_Demand (≈0.08). For P(Cost > 10%), Training_Level again has the highest EVPI (≈0.08), with Adoption_T3 close behind (≈0.05). These results indicate that even if technologies such as T1 and T3 are fully developed, uncertainty about training and client-driven requirements remains a major barrier to realizing their full potential benefits.

**Figure 5:**
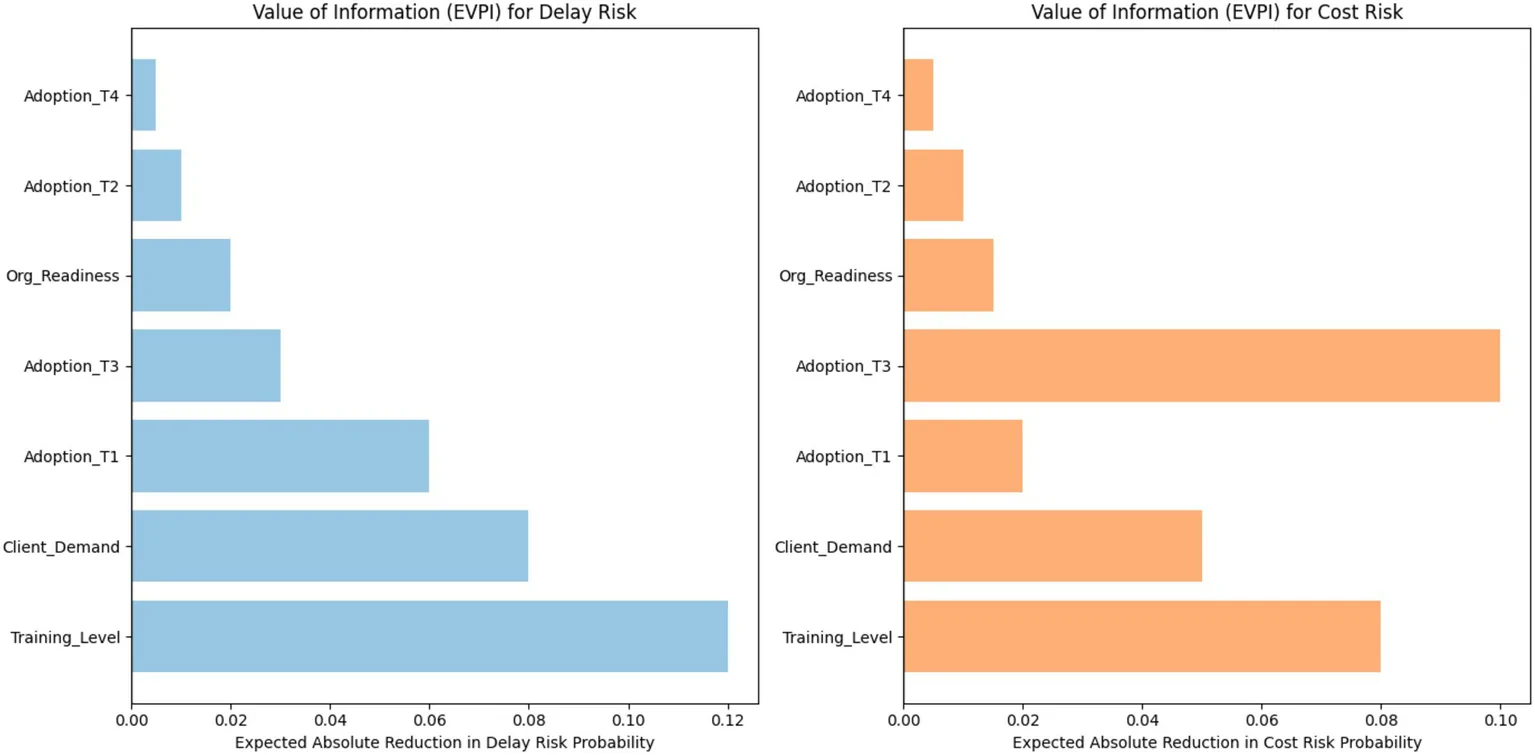
Expected Value of Perfect Information (EVPI) for selected context and technology-adoption nodes. The left panel shows the expected absolute reduction in P(Delay > 20%) if each variable were known with certainty, and the right panel shows the corresponding expected reduction in P(Cost overrun > 10%). EVPI values are reported as absolute probability-point reductions in the adverse outcome.

Figure 5 summarizes the EVPI values for key nodes, illustrating which uncertainties offer the greatest expected reduction in delay and cost risks if resolved.

### 4.5 Robustness and subgroup comparisons

Model robustness was investigated through the validation and sensitivity checks described in Section 3.7. Posterior predictive checks indicated good agreement between simulated and observed expert probabilities, and alternative expert-weighting schemes and slight perturbations to the BN structure did not affect the qualitative conclusions regarding influential factors or the relative impacts of the adoption scenarios. Priors on hierarchical hyperparameters also found to have limited influence, with posterior inferences dominated by the data.

Subgroup analyses provide additional insight into heterogeneity of expert beliefs. While all the professional groups are also in agreement on high baseline risks and substantial potential reduction under well-enabled technology adoption, contractors tend to report higher baseline probabilities of delay and cost overruns, reflecting a more conservative view consistent with direct exposure to delivery risks and penalties. Academics are somewhat more optimistic about the benefits of AI/BIM Digital Twins, likely reflecting greater familiarity with state-of-the-art digital tools and international evidence. Project managers and consultants tend to be between these views. Despite these variations in levels, all the groups agree that training and client demand, coupled with acceptance of T1 and T3, are key common levers in moving towards improvement in time and cost performance.

**Figure 6:**
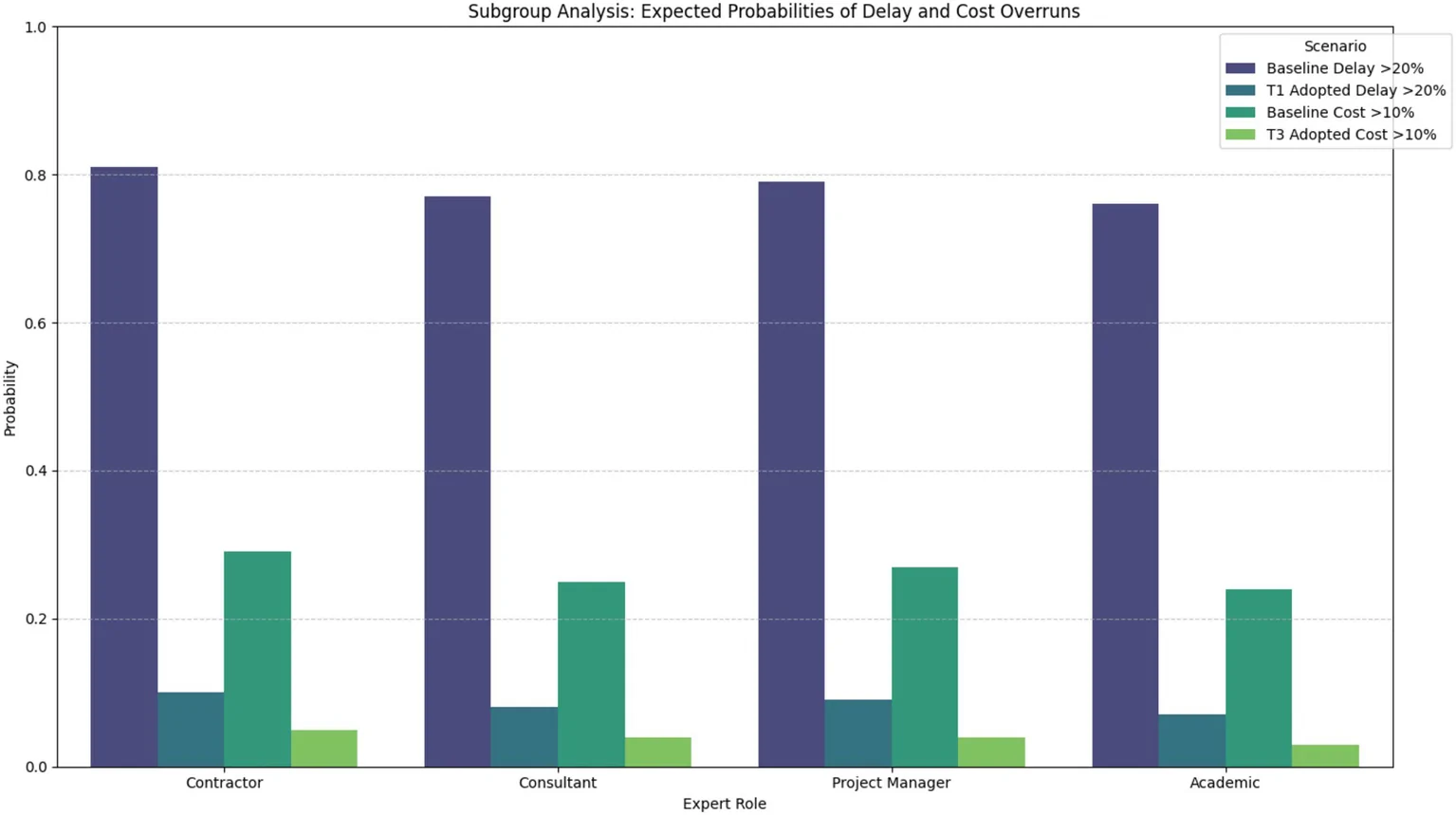
Subgroup comparison of expected probabilities P(Delay > 20%) and P(Cost > 10%) by dominant expert role (contractor, consultant, project manager, and academic) under baseline and technology-adopted scenarios. Scenario probabilities are derived from pooled expert judgements via the hierarchical Bayesian models and BN simulations.

Figure 6 shows the subgroup comparison of expected probabilities.

## 5 Discussion

This expert-elicited Bayesian Network presents a structured and uncertainty-aware perspective on the perceived impacts of AI-enabled BIM Digital Twin technologies on building-project performance in Jordan. This is a topic that remains sparsely covered in the digital-construction literature. The large baseline risk estimates (P(Delay > 20%) ≈ 0.78; P(Cost > 10%) ≈ 0.26) from all professional groups underline the extent to which schedule slippage and budget overruns have become the norm. These numbers are consistent with broader evidence from developing countries as well as with recent Jordan-specific studies on variation orders and project-performance risks (Abuassi et al., 2025; Bassam and Al-Btoush, 2026), which similarly portray chronic time–cost pressures in reinforced-concrete building projects. They are also broadly in line with international reports of frequent time–cost overruns in complex construction projects that rely on traditional management approaches (e.g., Khodakarami and Abdi, 2014; Kabir et al., 2018).

The improvements reported in this study are expectations derived from expert beliefs. They are not measured effects from implemented Digital Twin systems and should be interpreted as optimistic, upper-bound projections under mature, well-enabled adoption. Experts may therefore overestimate the reduction that can be realized in practice, especially in the short term and under less favourable implementation conditions. The model is therefore best viewed as a belief-based, uncertainty-aware decision-support tool, not as a fully validated forecasting model. All quantitative results come from structured expert judgement embedded in a Bayesian framework, rather than from longitudinal before–after project data. The marked reductions in risk under idealized scenarios—for example, a drop in P(Delay > 20%) from ≈0.78 to around 0.08 when T1 (AI-based progress detection) is assumed to be fully and effectively implemented—represent what experienced practitioners think might be achievable under highly favourable conditions. Compared with prior Bayesian-network applications to construction time–cost risk (e.g., Khodakarami and Abdi, 2014; Kabir et al., 2018), our BN formalizes large-scale expert beliefs about an emerging technology class rather than forecasting from project performance data. Empirical/pilot work on BIM/Digital Twins and AI-enabled monitoring reports improved visibility and earlier risk detection (e.g., Boje et al., 2020; Wang et al., 2024; Hsieh et al., 2026) but rarely provides comparable probabilistic effect sizes on delay/cost outcomes, especially in developing-country settings. We therefore report scenario outputs as optimistic, decision-relevant upper-bound expectations until local before–after evidence becomes available. They are not predictions of current Jordanian practice, but rather a belief-based state of mind that represents a range of possibilities based on high levels of training, client support and organizational readiness. In summary, our results are consistent with BN studies that highlight the value of better information and early risk detection (e.g., Khodakarami and Abdi, 2014; Kabir et al., 2018), and with Digital Twin case studies that report improved monitoring (Boje et al., 2020; Wang et al., 2024), but we extend this work by providing an explicit, quantified belief-based risk model for AI/BIM Digital Twins in a developing-country building context.

As a result, the magnitude of the reported risk reductions should be read as best-case beliefs, and actual realized impacts in early implementations are likely to be more modest.

Within this framing of belief, experts assign particularly strong potential to two clusters of technology. T1 (AI-based progress detection) is considered the key lever for achieving large reductions in schedule-overrun risk, and T3 (predictive cost–schedule Digital Twins) is considered the key lever for limiting budget overruns. These expectations broadly align with the aspirational benefits claimed in the international research on Digital Twins (Boje et al., 2020; Sacks et al., 2020; Eslahi et al., 2020; Wang et al., 2024), and with emerging empirical work on AI-driven progress monitoring and predictive planning (Hsieh et al., 2026; Pena et al., 2026; Liang et al., 2026). Existing BN-based project-risk models (e.g., Khodakarami and Abdi, 2014; Kabir et al., 2018) similarly demonstrate that improved information quality and early risk detection can, in principle, substantially reduce adverse outcomes. The contribution of the present study is to quantify this sector-level consensus in a developing-country context and to make explicit the causal pathways—through information accuracy, decision speed, coordination, design-change rates, and dispute propensity—by which these tools are believed to work.

One of the main findings of the analysis is that technology on its own is not the solution. The result of the global sensitivity analysis and value-of-information analysis is consistent for the impacts of Training Level and Client Demand on delay and cost risks, which are similar and, in some cases, larger than the adoption status of T1 and T3.

Scenarios in which AI/BIM Digital Twin tools are “switched on” but training is weak, and client requirements are modest, result in much smaller gains than scenarios where technology is combined with high human capability and clear client-side expectations. EVPI estimates reinforce this picture by identifying training and client demand as high-leverage uncertainties: if these were better understood and improved, expected project performance would shift substantially. The results are very similar to the socio-technical stories of BIM and digital innovation that focus on the importance of digital tools in enabling practices, contracts and regulatory frameworks (Chong et al., 2016; Tan et al., 2025). The subgroup comparisons provide additional nuance in the Jordanian context. Contractors, who are directly exposed to the risk of delay penalties and cost overruns, report somewhat higher baseline probabilities of both delay and cost overrun, reflecting a more conservative view of current performance. Academics, who tend to be closer to the latest digital-construction research and less constrained by day-to-day contractual pressures, are relatively more optimistic about the upside of AI/BIM Digital Twins. Consultants and project managers typically lie between these positions, balancing client demands, design-office practices, and site realities. Despite these differences in level, the qualitative patterns are strikingly consistent: all groups recognize (i) high current risks; (ii) potential for substantial risk reduction under high, well-enabled adoption; and (iii) the joint importance of training, client demand, and adoption of T1 and T3 as common levers for improving time and cost performance in Jordanian building projects.

Methodologically, the study contributes to the construction-management literature by moving beyond descriptive BIM-adoption surveys and isolated case studies toward a causal, probabilistic representation of expert knowledge. The hierarchical Bayesian structure is appropriate for aggregating heterogeneous judgements from 600 experts in different roles, allowing partial pooling and explicit quantification of uncertainty (Gelman et al., 2013). Integrating this with intervention analysis using a Bayesian Network makes it possible to examine policy-relevant “what-if” questions—such as introducing client-mandated progress-tracking tools, funding sector-wide training programs, or piloting predictive cost–schedule twins on public projects—within a coherent causal framework (Pearl, 2009; Kabir et al., 2018). Compared with earlier BN applications to cost and schedule risk (e.g., Khodakarami and Abdi, 2014) and to reliability and maintenance (Kabir et al., 2018), this study extends the approach to emerging AI/BIM Digital Twin technologies, explicitly models enabling conditions, and does so in a data-poor, developing-country setting.

In an environment such as Jordan where large-scale, high-quality before–after data on AI/BIM Digital Twin deployments are not yet available, a belief-based Bayesian approach can provide a realistic means of supporting early decision-making. The numerical outputs should not be interpreted as precise predictions, but as a disciplined, transparent synthesis of what a wide cross-section of Jordanian practitioners currently believe about the risks and opportunities of AI-enabled BIM Digital Twins. As empirical evidence accumulates from pilot projects and broader implementations, the same modelling framework can be updated to incorporate observed performance data and gradually reduce reliance on purely subjective judgements. A key next step is therefore to validate and recalibrate the Bayesian Network using real-world project datasets from AI/BIM Digital Twin deployments in Jordan. This evolution—from expert-only to mixed expert-and-data-driven inference—would align with broader practice in Bayesian risk modelling and could ultimately provide more robust, empirically grounded decision support for policy makers, clients, and industry leaders.

## 6 Conclusion

This study created an expert elicitation process for a Bayesian Network to quantify the time and cost risks where AI-enabled BIM Digital Twin technologies are foreseen to impact building projects in Jordan. Based on structured judgments involving 600 different contractors, consultants, project managers, and academics, it formalized a causal representation of interactions among four technology types, which are AI-based progress detection (T1), contract-aware BIM assistants (T2), predictive cost schedule twins (T3), and AI-assisted design/constructability (T4), by contextual enablers and intermediate project mechanisms.

Using hierarchical Bayesian inference on elicited probabilities, we estimated pooled baseline and technology conditional risks and embedded the estimates in a Bayesian Network supporting uncertainty-aware scenario analysis. Experts have high baseline risks (median P(Delay > 20%) ≈ 0.78; median P(Cost > 10%) ≈ 0.26) and very substantial potential reductions under optimistic scenarios of full adoption combined with strong organizational readiness (P(Delay > 20%) ≈ 0.08–0.10 with effective T1; P(Cost > 10%) ≈ 0.03–0.05 with effective T3). These are not measurements from projects but for what informed practitioners feel is achievable in a mature and well-supported digital construction environment.

The scenario, sensitivity and value-of-information analyses together suggest that digital technologies must be accompanied by enabling conditions if they are to reduce time and cost risks meaningfully. Over and over again, training level and client demand turn up as important moderating variables, with influence that sometimes looks comparable to the adoption status of T1 and T3. Scenarios involving high levels of technologies and low levels of training or weak client requirements yield low levels of benefits. Still, combinations of technology, skills and clear client/regulatory mandates yield the largest reductions in predicted risks. This highlights the importance of integrated approaches that involve both investment in AI/BIM Digital Twin technology and systematic capacity building and support systems, commensurate with effective procurement and regulatory policies.

Three contributions of this study are made. First, it presents the first formal, expert-based causal structure between AI-enabled BIM Digital Twins and time and cost performance in Jordanian building construction. Second, it provides a quantified and uncertainty-aware Bayesian Network, parameterized using hierarchical Bayesian pooling, which could be utilized for the exploration of alternative adoption and policy scenarios in a transparent manner. Third, it produces policy-relevant insights regarding the relative leverage of technology adoption, training and client/regulatory requirements for moving digital transformation forward in a developing-country construction context.

Because the analysis is entirely based on expert elicitation, the numerical results should be considered conditional expectations and not empirically tested effects. The future research should focus on the empirical validation of the technologies through pilot projects with the implementation of selected technologies and the concomitant systematic collection of baseline and post-adoption time–cost performance data. Such data would allow formal validation and refinement of the current expert-elicited Bayesian Network against observed time–cost outcomes. Such evidence would allow for progressive updates of the Bayesian Network, the evolution of the methodology to other types of assets and performance dimensions (e.g., safety, quality, and sustainability), as well as comparative applications in other countries. More broadly, the study is important in demonstrating how structured expert elicitation coupled with Bayesian Network modelling could offer an early decision support tool for uncertainty-aware considerations of emerging digital technologies in data-poor construction environments.

### 6.1 Limitations and future research

This study has several limitations that should be borne in mind when interpreting the results and which point to directions for further work.

First, all quantitative findings in this study are based on structured expert judgement, not on observed before–after project performance data. The Bayesian Network is therefore a belief-based representation of expert views, not an empirically validated predictive model. The reported probabilities and risk reductions are conditional expectations based on pooled expert beliefs, rather than identified causal effects or externally validated forecasts. Although the protocol for eliciting responses, a rather large expert sample (*N* = 600), and hierarchical pooling minimize any one individual’s biases, perhaps overconfidence, salient project anchoring, or strategic optimism/pessimism still may be present. In addition, snowball/network recruitment may over-represent digitally oriented practitioners, which could bias scenario benefit upward and limit generalizability to less digital firms and project settings.

External validity is limited: elicited beliefs may not generalize across firms, project types, or the future implementation conditions in Jordan.

In particular, the large reductions in delay and cost risks under the idealized high-adoption scenarios should be regarded as potentially optimistic, conditional upper limits rather than as guaranteed or typical outcomes.

Second, the four technology clusters (T1-T4) and the Bayesian network structure obviously simplify a heterogeneous and changing technology landscape. Different AI/BIM tools, implementation strategies, types of contracts, and firm capabilities are aggregated into a handful of abstract nodes. The model also compresses variation between types of buildings, contractual arrangements, and market segments in Jordan, and does not explicitly model dynamic phenomena such as learning curves, phased rollout, or feedback between perceived success and subsequent adoption.

Third, the magnitude of the predicted risk reductions assuming ideal conditions should be considered as aspirational, conditional upper limits. The scenarios are set up to examine long-term possibilities under high levels of adoption, training, and organizational readiness, not to forecast near-term impacts of current pilot projects. The analysis does not contain explicit cost–benefit analysis of technology investments, nor is the implementation of these technologies quantified by considerations such as poor data quality, vendor lock-in, cybersecurity concerns, or organizational resistance, other than in their indirect representation via high-level context nodes.

Thus, economic viability and return on investment are not assessed in this study.

Fourth, although we investigated alternative schemes for weighting expertise, slight structural perturbations of the Bayesian Network, or slight perturbations of prior specifications, we did not conduct formal model comparisons among competing network structures or among more complex functional forms of context effects. More systematic application of algorithms based on learning of structure, model averaging or cross-validation against emerging empirical data could also provide further strength to the robustness of the framework.

These limitations indicate several directions for the future research. On the empirical side, there exists an apparent need for longitudinal pilot studies in Jordanian building projects that will employ certain AI/BIM and Digital Twins solutions (e.g., T1 and T3) and will systematically track time–cost performance before and after application. Such data would allow calibrated information to be provided to the existing Bayesian Network and updated, gradually reducing the balance to favour subjective inference more and more and also mixed subjective-empirical inference. Methodologically speaking, the framework could be expanded to reflect other dimensions of performance (such as safety, quality, carbon emission, and energy performances), more detailed project typologies, and dynamic adoption processes over several project cycles. Comparative applications in other countries and sectors (e.g., infrastructure and industrial facilities) would help differentiate between context-invariant mechanisms and those specific to the Jordanian institutional and market environment. Finally, incorporating explicit economic evaluation (e.g., net present value analysis of various combinations of technology adoption, training investments and client/regulatory interventions) with the Bayesian Network-based decision framework would provide more comprehensive guidance for policy makers and industry leaders as they consider large-scale investment in AI-enabled BIM Digital Twins.

## Data Availability

The raw data supporting the conclusions of this article will be made available by the authors, without undue reservation.
