# Supplementary material for: Assessing the impact of AI-enabled BIM digital twins on construction risks: a Bayesian Network of Jordanian expert beliefs
Source: Front Artif Intell. 2026 Aug 25;9:1846388. doi: 10.3389/frai.2026.1846388 (PMC13550160; doi:10.3389/frai.2026.1846388)
Supplement: Supplementary file 1 [file Data_Sheet_1.DOCX]

# Supplementary Material S1 — Full Interview Script and Questionnaire (Expert Elicitation Instrument)Interview Instrument

## AI-Enabled BIM Digital Twin Technologies and Construction Management in Jordan

# Participant Consent Statement

“This interview seeks professional opinions regarding AI-enabled BIM digital twin technologies and construction management practices. Participation is entirely voluntary, and you may withdraw at any time without consequence. All responses will remain anonymous and will be used solely for academic research purposes.

Do you agree to participate in this interview?”

**Consent:** ☐ Yes ☐ No
*(If No → End interview)*

# Section 1 — Probability Guidance (Brief Training)

### Instruction to Participant

“For the following probability-related questions, please provide a single percentage value ranging from 0% to 100%. If you are uncertain, please provide your best estimate based on your professional experience.”

### Interviewer Guidance

- If the participant hesitates: *“Please provide your best single-number estimate and think about projects you are familiar with.”*
- If the participant answers 0% or 100%: *“Does this represent complete certainty, or would you like to reconsider your estimate?”*

# Section 2 — Core Probability Assessment (Time and Cost Risks)

## Q2.1 Baseline Schedule Risk (Current Practice)

Under current construction practices in Jordan, without advanced AI/BIM digital twin technologies, what is the probability that a project will experience a schedule delay exceeding **20%** of the planned duration?

Response

**P(Delay > 20%

## Q2.2 Technology-Conditional Schedule Risk (T1: Effective AI-Based Progress Detection)

Consider the same project scenario where AI-based progress detection technologies (T1) are widely adopted and effectively implemented, supported by strong organizational readiness and adequate training.

What is the probability that the project will experience a schedule delay exceeding **20%**?

Response

**P(Delay > 20%

## Q2.3 Baseline Cost Risk (Current Practice)

Under current construction practices in Jordan, what is the probability that a project will experience a cost overrun exceeding **10%** of the planned budget?

Response

**P(Cost > 10%

## Q2.4 Technology-Conditional Cost Risk (T3: Predictive Cost–Schedule Digital Twin)

Consider the same project scenario where predictive cost–schedule digital twin technologies (T3) are widely adopted and effectively implemented, supported by strong organizational readiness and adequate training.

What is the probability that the project will experience a cost overrun exceeding **10%**?

Response

**P(Cost > 10%

# Section 3 — Causal Link and Impact Ratings

### Rating Scale

| Scale | Meaning |
| --- | --- |
| 1 | No Effect |
| 2 | Weak Effect |
| 3 | Moderate Effect |
| 4 | Strong Effect |
| 5 | Very Strong Positive Effect |

Please select one response for each statement.

## T1 — AI-Based Progress Detection

| Question Code | Statement | 1 | 2 | 3 | 4 | 5 |
| --- | --- | --- | --- | --- | --- | --- |
| Q3.1 | Effective use of T1 increases on-site information accuracy. | ☐ | ☐ | ☐ | ☐ | ☐ |
| Q3.2 | Effective use of T1 improves schedule control (reducing delays). | ☐ | ☐ | ☐ | ☐ | ☐ |
| Q3.3 | Effective use of T1 improves cost control (reducing overruns). | ☐ | ☐ | ☐ | ☐ | ☐ |

## T2 — Contract-Aware BIM Assistants

| Question Code | Statement | 1 | 2 | 3 | 4 | 5 |
| --- | --- | --- | --- | --- | --- | --- |
| Q3.4 | Effective use of contract-aware BIM assistants (T2) reduces claims and disputes. | ☐ | ☐ | ☐ | ☐ | ☐ |

## T3 — Predictive Cost–Schedule Digital Twin

| Question Code | Statement | 1 | 2 | 3 | 4 | 5 |
| --- | --- | --- | --- | --- | --- | --- |
| Q3.5 | Effective use of predictive cost–schedule digital twins (T3) improves early detection of time and cost risks. | ☐ | ☐ | ☐ | ☐ | ☐ |

## T4 — AI-Assisted Design and Constructability

| Question Code | Statement | 1 | 2 | 3 | 4 | 5 |
| --- | --- | --- | --- | --- | --- | --- |
| Q3.6 | Effective use of AI-assisted design and constructability tools (T4) reduces design changes during construction. | ☐ | ☐ | ☐ | ☐ | ☐ |

# Section 4 — Context and Enablers

## Q4.1 Training Level

Select One

☐ Low

☐ Medium

☐ High

## Q4.2 Client Demand

Select One

☐ Low

☐ Medium

☐ High

## Q4.3 Organizational Readiness

Select One

☐ Low

☐ Medium

☐ High

## Q4.4 Effect of Poor Training and Organizational Readiness

“If training and organizational readiness are poor, the benefits of AI/BIM digital twin technologies are most likely:”

Select One

☐ Almost no benefit remains

☐ Benefits reduced by approximately 75%

☐ Benefits reduced by approximately 50%

☐ Benefits reduced by approximately 25%

☐ Benefits remain almost the same as with good training

# Section 5 — Barriers and Accelerators

## Q5.1 Main Barrier to Adoption

Select One

☐ Lack of client demand

☐ Lack of standards

☐ High cost of tools

☐ Lack of skills

☐ Resistance to change

## Q5.2 Main Accelerator for Adoption

Select One

☐ Strong client requirements

☐ Government / BIM mandate

☐ Subsidised training

☐ Success stories from major projects

☐ Vendor support

# Section 6 — Respondent Metadata

## Q6.1 Dominant Professional Role

Select One

☐ Contractor

☐ Consultant

☐ Project Manager

☐ Academic

## Q6.2 Experience Category

Select One

☐ Less than 5 years

☐ 5–10 years

☐ 11–20 years

☐ More than 20 years

## Q6.3 BIM Use Frequency

Select One

☐ Never

☐ Occasionally

☐ Frequently

☐ Almost always

## Q6.4 Self-Rated BIM Skill Level

| 1 | 2 | 3 | 4 | 5 |
| --- | --- | --- | --- | --- |
| ☐ | ☐ | ☐ | ☐ | ☐ |

## Q6.5 Interview Mode

Select One

☐ Telephone

☐ Zoom

☐ Face-to-face

# Closing Statement

“Thank you for your participation. This completes the interview. Your responses will remain anonymous and will be used solely for academic research purposes.”
